# Remimazolam anesthesia for cardiac surgery with cardiopulmonary bypass: a case report

**DOI:** 10.1186/s40981-021-00424-0

**Published:** 2021-03-06

**Authors:** Kota Saito, Sho Ohno, Makishi Maeda, Naoyuki Hirata, Michiaki Yamakage

**Affiliations:** Department of Anesthesiology, School of Medicine, Sapporo Medical University, South 1, West 16, Chuo-ku, Sapporo, Hokkaido 060-8543 Japan

**Keywords:** Remimazolam, Cardiac surgery, Cardiopulmonary bypass

## Abstract

**Background:**

Remimazolam has less cardiovascular depressant effects than propofol in non-cardiac surgical patients. However, the efficacy and safety of remimazolam in cardiac surgery with cardiopulmonary bypass (CPB) have not been reported. We present a case of successful anesthetic management using remimazolam in cardiac surgery with CPB.

**Case presentation:**

A 76-year-old female was scheduled for mitral valve repair, tricuspid annuloplasty, maze procedure, and left atrial appendage closure. We used remimazolam in induction (6.0 mg/kg/h) and maintenance (0.6–1.0 mg/kg/h) of general anesthesia, and the bispectral index value was maintained in the range of 36 to 48 including the period of CPB. Hemodynamics, mixed venous oxygen saturation, and bilateral regional cerebral oxygen saturation were maintained within acceptable ranges. There was no intraoperative awareness/recall or serious complications associated with remimazolam throughout the perioperative period.

**Conclusions:**

Remimazolam can be used the same as other existing anesthetics in cardiac surgery with CPB.

## Background

Remimazolam is a new ultra-short-acting benzodiazepine sedative, and its simulated context-sensitive half-time after infusion for 4 h is 6.8 ± 2.4 min [1]. Remimazolam is equally effective as propofol for induction and maintenance of general anesthesia [2]. It has been reported that benzodiazepine sedatives have less cardiovascular depressant effects in cardiac surgery [3] and that flumazenil can antagonize the action of benzodiazepine sedatives [4]. In addition, remimazolam causes no pain at the injection site [5]. Patients undergoing cardiac surgery are vulnerable to hemodynamic instability such as hypotension, especially during the induction of anesthesia, and anesthesiologists should therefore administer anesthetic agents carefully to avoid cardiovascular depressant effects. Considering its advantage of less cardiovascular depressant effect, remimazolam might be a better sedative during anesthetic management of cardiovascular surgery. However, patients undergoing cardiac surgery with cardiopulmonary bypass (CPB) have been excluded in phase IIb/III clinical trials [2, 5]. Thus, general anesthetic management with remimazolam in cardiac surgery, especially with CPB, has not been reported.

In this case report, we present a case in which remimazolam was used in cardiac surgery with CPB.

## Case presentation

Approval and written informed consent for publication of this report were obtained from the patient.

A 76-year-old female (height, 155 cm; weight, 59.6 kg) had chronic heart failure with severe mitral regurgitation (MR), mild tricuspid regurgitation (TR), and chronical atrial fibrillation. Her comorbidities included hypertension and old cerebral infarction at the right medulla oblongata. A preoperative transthoracic echocardiogram (TTE) showed a left ventricular ejection fraction of 62% and no left ventricular wall asynergy. Mitral valve repair, tricuspid annuloplasty, maze procedure, and left atrial appendage closure were scheduled. The surgical risk calculated by the Society of Thoracic Surgeons score (STS score) was 2.23% and that calculated by the European System for Cardiac Operative Risk Evaluation (EURO score) was 2.52%.

**Fig. 1:**
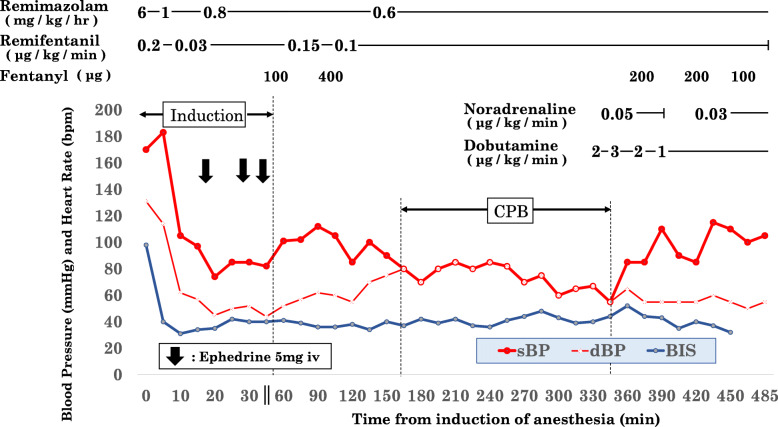
Anesthetic record. sBP, systolic blood pressure; dBP, diastolic blood pressure; BIS, bispectal index

On arrival at the operating room, we inserted an arterial line into the right radial artery to monitor hemodynamics. And we also monitored bilateral regional cerebral oxygen saturation (rSO_2_) by using INVOS^TM^ and the bispectal index (BIS) value to assess the patient’s depth of sedation. Anesthesia was induced using remimazolam at 6 mg/kg/h, remifentanil at 0.25 μg/kg/min, and rocuronium at 0.85 mg/kg. The patient did not complain of pain at the infusion site during administration of remimazolam. Remimazolam was adjusted by 1 mg/kg/h after loss of consciousness (LoC). The time from administration of remimazolam to LoC was 130 s. We inserted central venous and pulmonary artery catheters from the right internal jugular vein as well as a probe for monitoring transesophageal echocardiography (TEE) after intubation. During induction, the patient’s mean blood pressure (BP) decreased by more than 50%, from 137 to 54 mmHg, and 5 mg of ephedrine was administered three times to maintain mean BP over 65 mmHg. rSO_2_ was decreased within 10% from the baseline value regardless of hypotension during induction. The BIS value ranged from 31 to 42 in the period of induction (Fig. 1). Remimazolam was adjusted by 0.6–1.0 mg/kg/h according to the BIS value until the end of the surgery, and the depth of sedation was stable when the BIS value ranged from 30 to 50, even in the period of undergoing CPB. We did not induce hypothermia, and body temperature was maintained at 35–36 °C during CPB.

Surgical procedures were completed successfully. While left ventricular function was preserved after CPB, oxygenation status deteriorated after CPB. PaO_2_/FiO_2_ ratio (P/F ratio) decreased to 104 mmHg from 250 mmHg before CPB. Although we repeated sputum suction and recruitment maneuvers, P/F ratio was not improved (136 mmHg) at discharge from the operating room. TEE showed trivial MR remaining after mitral valve repair and systolic function was preserved. At the end of the surgery, the intraoperative dose of fentanyl was 17 μg/kg. Intraoperative infusion volume was 1300 ml and we transfused 280 ml of red blood cells and 720 ml of fresh frozen plasma. Bleeding volume was 360 ml, and water balance was + 1570 ml in total. Total operation time was 365 min, total anesthesia time was 474 min, and CPB time was 252 min.

We transferred the patient from the operating room to the intensive care unit (ICU). Administration of remimazolam was stopped after arriving at the ICU. A chest X-ray and ultrasonography showed mild pleural effusion and atelectasis in the right lung field. We did not antagonize remimazolam with flumazenil because of the poor oxygenation. Dexmedetomidine was administered until her oxygenation improved sufficiently for extubation. The patient showed a nodding response about 90 min after arriving at the ICU. The tracheal tube was removed 220 min later when the P/F ratio was 248 mmHg. We confirmed that there was no intraoperative awareness or recall and that there were no serious complications associated with the use of remimazolam throughout the perioperative period. The patient was moved to the general ward on postoperative day (POD) 1. The patient had disorientation and delirium on POD 6, but magnetic resonance imaging (MRI) showed no new lesion of cerebral infarction. The patient showed a motor and sensory disorder in her left lower limb on POD 10 and she was diagnosed with right cerebral infarction. She was discharged on POD 41 and transferred to another hospital to continue rehabilitation.

## Discussion

In the management of general anesthesia in cardiac surgery, it is important to maintain hemodynamic stability and avoid a reduction in mean BP, especially during induction of anesthesia [6]. Propofol is commonly used as a sedative for intravenous anesthesia in induction and maintenance of anesthesia. However, the degree of BP reduction in induction is mild when benzodiazepine sedatives are used in cardiac surgery [3]. Therefore, midazolam, one of the benzodiazepine sedatives, is also used in induction but not in maintenance of anesthesia because its continuous administration could cause awaking delay due to its long half-life and accumulation [7]. In addition, it has been reported that fast-track surgery including early extubation shortened the period of ICU stay and saved medical resources [8]. For this reason, early and clear awareness is required for extubation in the ICU after a cardiac operation under certain conditions such as good cardiac function and sufficient hemostasis.

In the present case, there was quite a large decrease in mean BP from baseline BP, from 137 to 54 mmHg, but BP soon recovered with ephedrine administration in the period of anesthesia induction. Although the recommended dose is 12 mg/kg/h in induction, we administered 6 mg/kg/h for safety considering the patient’s cardiac condition. Throughout anesthesia, rSO_2_ was maintained within 10% from the baseline value. Administration of a low dose of catecholamine, dobutamine (2.0 μg/kg/min), and noradrenaline (0.03 μg/kg/min) enabled hemodynamics to be maintained after CPB. Further study is required to determine the suitable dose of remimazolam for the less cardiovascular depressant effect in cardiac surgery.

Sedation of the patient was continued after surgery because of the low oxygenation status. However, there is a possibility that anesthesiologists might be able to achieve earlier recovery from anesthesia and assess the patient’s neurological signs early after surgery through the short-acting characteristics of remimazolam and antagonizing with flumazenil.

We also used remimazolam during maintenance of anesthesia, even in the period of CPB, based on BIS values. Sheng et al. reported that BIS monitoring is appropriate for assessing awareness signs during remimazolam anesthesia [9]. Although we did not administer any additional sedatives, the patient did not have any intraoperative recall/awareness. We believe that BIS monitoring enables assessment of the depth of sedation in remimazolam anesthesia.

Previous studies showed that while the concentration of propofol is diluted by the priming volume during CPB, the necessary dose of propofol tends not to be increased for the following reasons. First, the free drug concentration of propofol increases with decrease in the plasma albumin level [10]. Second, hypothermia and decreased hepatic perfusion possibly make propofol metabolism slower [11]. In addition, it is well known that some sedative agents can be absorbed by CPB devices [12]. On the other hand, remimazolam is metabolized by elastase, which is produced by the liver, and it is thought to be metabolized earlier compared to other existing anesthetic agents [13]. Considering the changes in the BIS value during CPB, remimazolam might have a tendency similar to that of propofol regarding the necessary dose during CPB. To reveal this, detailed pharmacokinetic and pharmacodynamics analyses of remimazolam during CPB would be required for the safe use of remimazolam in cardiac surgery.

In summary, we could use remimazolam in induction and maintenance of anesthesia for cardiac surgery with CPB. Considering the advantages of benzodiazepine sedatives as mentioned above and the ultra-short-acting property of remimazolam, remimazolam has the possibility of being a novel sedative in the field of cardiovascular anesthesia. Further research is necessary to reveal its effectiveness and disadvantages by using remimazolam in various cases.

## Data Availability

Not applicable

## Abbreviations

CPB: Cardiopulmonary bypass
MR: Mitral regurgitation
TR: Tricuspid regurgitation
TTE: Transthoracic echocardiogram
TEE: Transesophageal echocardiography
rSO_2_: Bilateral regional cerebral oxygen saturation
BP: Blood pressure
BIS: Bispectal index
LoC: Loss of consciousness
P/F ratio: PaO_2_/FiO_2_ ratio
ICU: Intensive care unit
MRI: Magnetic resonance imaging
PCPS: Percutaneous cardiopulmonary support
